# Femoral vein occlusion after VASCADE MVP resolved without surgery: a case report

**DOI:** 10.1093/ehjcr/ytaf581

**Published:** 2025-11-08

**Authors:** Masaki Honda, Masateru Takigawa, Taishi Yonetsu, Shinsuke Miyazaki, Tetsuo Sasano

**Affiliations:** Department of Cardiovascular Medicine, Institute of Science Tokyo, Yushima 1-5-45, Bunkyo-ku, Tokyo 113-8510, Japan; Department of Cardiovascular Medicine, Institute of Science Tokyo, Yushima 1-5-45, Bunkyo-ku, Tokyo 113-8510, Japan; Department of Cardiovascular Medicine, Institute of Science Tokyo, Yushima 1-5-45, Bunkyo-ku, Tokyo 113-8510, Japan; Department of Cardiovascular Medicine, Institute of Science Tokyo, Yushima 1-5-45, Bunkyo-ku, Tokyo 113-8510, Japan; Department of Cardiovascular Medicine, Institute of Science Tokyo, Yushima 1-5-45, Bunkyo-ku, Tokyo 113-8510, Japan

**Keywords:** VASCADE MVP, Femoral vein occlusion, Collagen plug, Endovascular treatment, Intravascular endoscopy, Case report

## Abstract

**Background:**

VASCADE MVP® is a vascular closure device developed to achieve haemostasis of large-bore venous access by deploying a collagen patch in the subcutaneous tissue. While its efficacy and safety have been demonstrated, venous occlusion has not been previously reported.

**Case summary:**

A 72-year-old woman was hospitalized and underwent pulmonary vein isolation for symptomatic paroxysmal atrial fibrillation. Two femoral venous sheaths (10-Fr and 8.5-Fr) were inserted under ultrasound guidance, and haemostasis was achieved using VASCADE MVP Venous Vascular Closure devices. On postoperative day 9, right leg swelling appeared. Contrast-enhanced CT showed stenosis of the right common femoral vein (CFV), and duplex ultrasound revealed a hyperechoic structure distal to the stenosis. Since deep vein thrombosis was suspected, the apixaban dose was adjusted from 10 to 20 mg/day. Due to persistent symptoms, venography was performed on Day 15, which revealed complete CFV occlusion. Intravascular endoscopy revealed white intraluminal material consistent with residual collagen, and subsequent balloon angioplasty led to partial restoration of blood flow. Given the gradual improvement in symptoms, conservative management was pursued, leading to complete recovery at 2 months as confirmed by duplex ultrasound.

**Discussion:**

This is the first reported case of venous occlusion likely caused by residual collagen from a collagen-based venous closure device. Balloon angioplasty restored partial blood flow, and surgical intervention was avoided due to the identification of collagen-induced reversible mechanical obstruction. When using VASCADE MVP, operators should be mindful to avoid intravascular deployment of collagen by ensuring proper technique.

Learning points

- Residual collagen from a vascular closure device can cause non-thrombotic mechanical venous occlusion.
- Endovascular treatment enabled identification of the obstruction site and partial restoration of flow, which potentially avoided surgical intervention.

## Introduction

VASCADE MVP^®^ (Cardiva Medical, a part of Haemonetics, Natick, MA, USA) is a vascular closure device specifically designed for achieving haemostasis after the removal of large-bore venous sheaths, such as those used in catheter ablation procedures. The device deploys an absorbable collagen patch into the extravascular tissue. Its efficacy and safety were demonstrated in the randomized AMBULATE trial, which showed reductions in procedure time, time to haemostasis, hospital stay, and a low incidence of complications.^1^

## Summary figure

**Figure:**
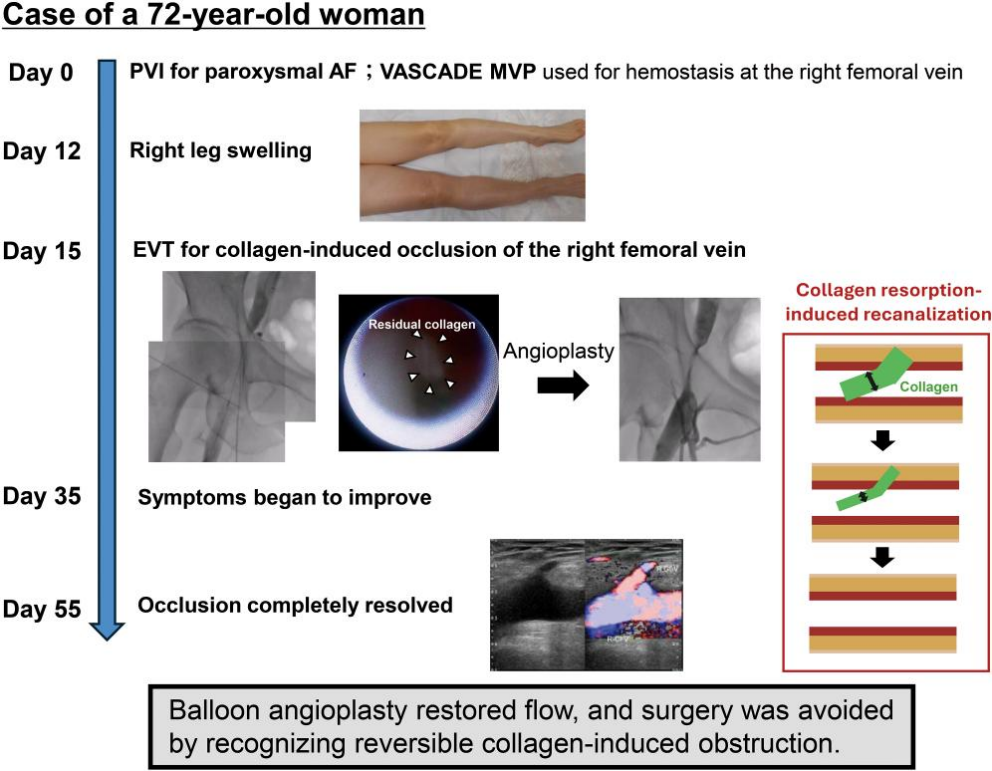


Suture-mediated closure devices such as Perclose ProGlide® are also widely used for femoral venous haemostasis after electrophysiology procedures. Reports have described complications, including venous occlusion that occasionally required surgical intervention.^2–4^ In contrast, no published cases of venous occlusion caused by collagen-based devices such as VASCADE MVP have been reported. Registry data from the AMBULATE SDD study and analyses of the US Food and Drug Administration (FDA) Manufacturer and User Facility Device Experience (MAUDE) database further support the overall safety of VASCADE MVP with no signal of venous occlusion.^5,6^ Nevertheless, no peer-reviewed head-to-head studies directly comparing Perclose and VASCADE MVP are available; therefore, it cannot be concluded which closure device is safer or more effective. What is essential is that both devices be used with careful attention to safety. Here, we present the first reported case of reversible venous occlusion associated with VASCADE MVP, which we believe may contribute to safer use of venous closure devices in the future.

## Case presentation

A 72-year-old woman (height 158.5 cm, weight 56.7 kg, BMI 22.7) with symptomatic paroxysmal atrial fibrillation was admitted for pulmonary vein isolation. She was taking apixaban 10 mg/day. Under ultrasound guidance, two sheaths—a 10-Fr sheath and an 8.5-Fr sheath—were inserted via the right femoral vein. Haemostasis was achieved with two VASCADE MVP Venous Vascular Closure devices.

**Figure 1:**
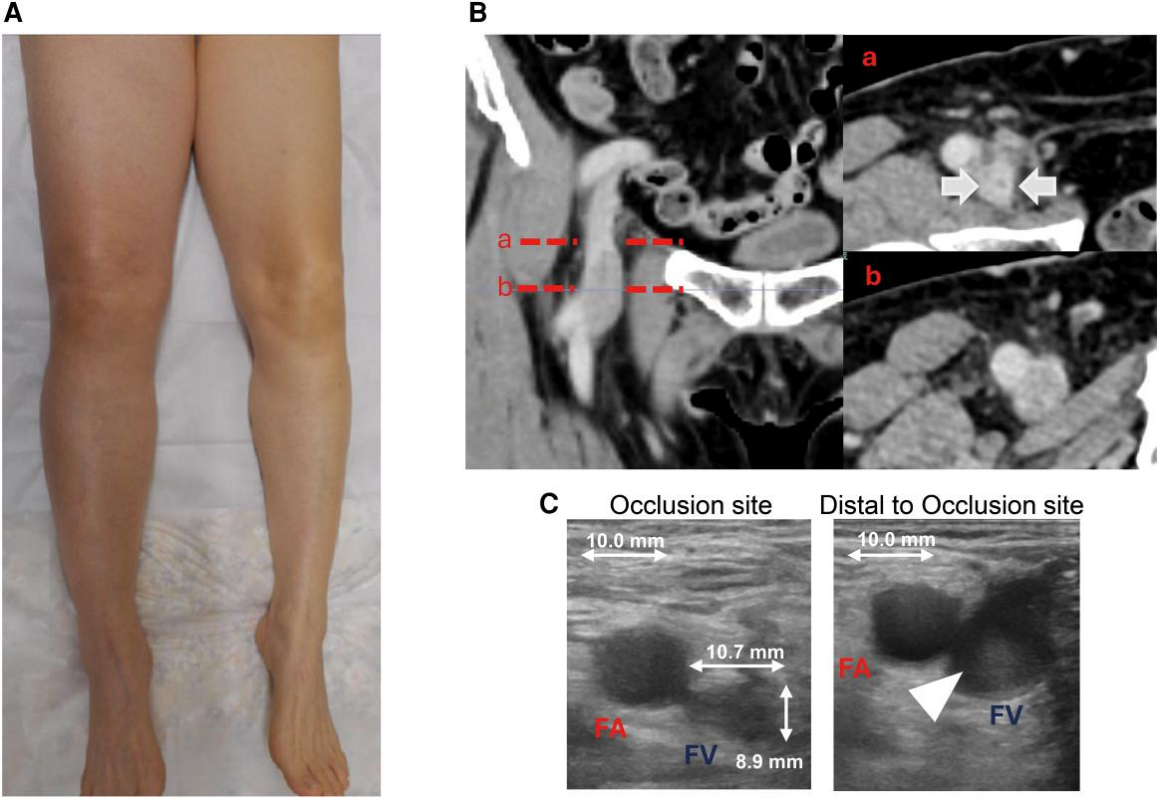
Clinical and imaging findings on Day 12. (*A*) Physical examination showing diffuse swelling and mild erythema in the right lower extremity. (*B*) Contrast-enhanced computed tomography (CT) demonstrating narrowing of the right common femoral vein (CFV) with peripheral venous dilation. Axial slices at two levels (a, b) are shown on the right. White arrows indicate the narrowed CFV lumen. (*C*) Duplex ultrasonography showing narrowing of the right CFV. The left panel (occlusion site) demonstrates the vessel dimensions (10.9 mm × 9.4 mm), while the right panel (distal to the occlusion site) reveals a hyperechoic lesion (arrowhead) within the vein, suggestive of thrombus. FA, femoral artery; FV, femoral vein.

**Figure 2:**
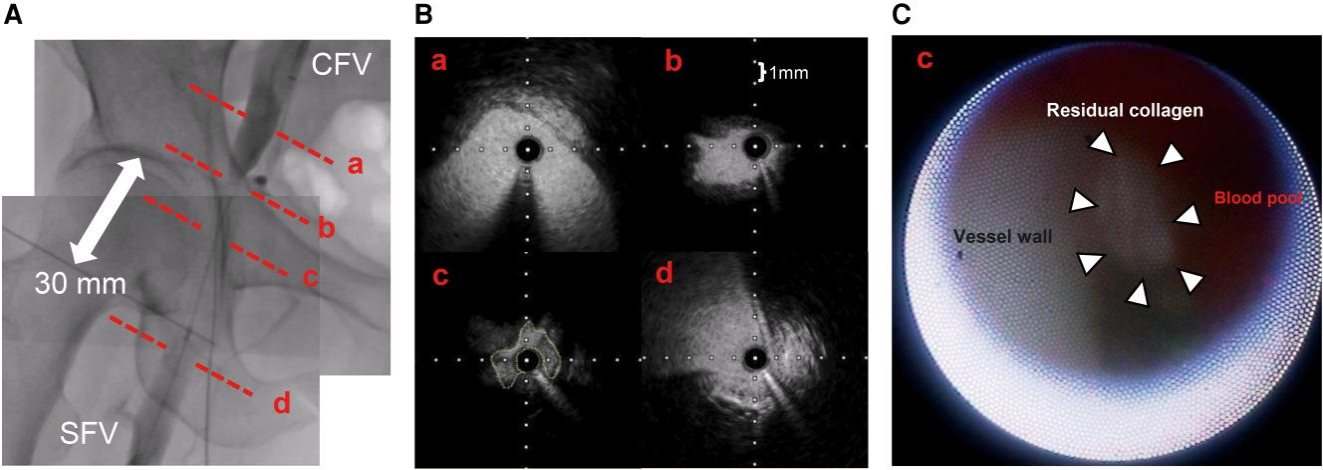
Multimodality imaging before endovascular treatment. (*A*) Composite venography before balloon angioplasty. Images from both proximal and distal contrast injections were merged. A total occlusion of the right common femoral vein (CFV), ∼30 mm in length, was observed (white arrow). Red dashed lines (a–d) represent the cross-sectional positions of the IVUS images in panel B; among them, point c corresponds to the site visualized by intravascular endoscopy in panel C. (*B*) Intravascular ultrasound (IVUS) images corresponding to levels a–d in panel A. a: healthy segment proximal to the lesion. b: segment just proximal to the lesion, showing slight venous wall shrinkage. c: site of intraluminal obstruction. A medium echogenic structure compressing the vessel lumen is observed from the 7 to 4 o’clock direction, outlined in green, and presumed to be residual collagen. d: healthy segment distal to the lesion. (*C*) Intravascular endoscopy at level c, showing a white intraluminal material (white arrowheads), presumed to be residual collagen from the VASCADE MVP device. The vessel wall and a pool of blood are visible proximal to the material. SFV, superficial femoral vein.

The patient was discharged on Day 2 without swelling in her lower extremities. However, swelling of the right lower limb developed on Day 9 and progressively worsened, leading to an unplanned outpatient visit on Day 12. Physical examination revealed diffuse swelling and mild erythema in the right lower extremity (*Figure 1A*). Contrast-enhanced computed tomography (CT) showed narrowing of the right common femoral vein (CFV), peripheral venous dilation, and no evidence of pulmonary embolism (*Figure 1B*). Duplex ultrasonography demonstrated narrowing of the CFV and a hyperechoic lesion within the vein distal to the stenotic segment (*Figure 1C*). The vessel diameter at the occluded segment measured 10.9 mm × 9.4 mm. Although the D-dimer level was negative, deep vein thrombosis was suspected based on the ultrasonographic findings. Symptoms did not improve, and venography on Day 15 revealed a total occlusion of the right CFV, ∼30 mm in length, from the upper margin of the femoral head (*Figure 2A*, *Video 1*, Supplementary material online, *Video S1*).

**Figure 3:**
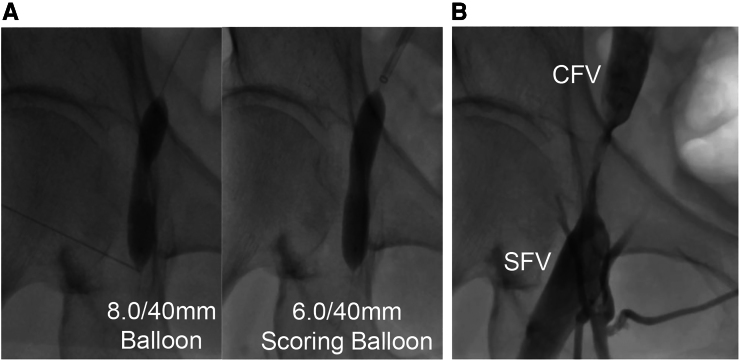
Balloon angioplasty for common femoral vein occlusion. (*A*) Balloon dilation with 8.0/40 mm balloon (left) and 6.0/40 mm scoring balloon (right). Indentation remained on both balloons during inflation, and the patient reported localized venous pain during the procedure. (*B*) Post-angioplasty venography showing partial contrast passage across the residual severe stenosis, estimated at ∼90%. CFV, common femoral vein; SFV, superficial femoral vein.

Intravascular ultrasound (IVUS) showed an isoechoic protruding mass accompanied by echo signal attenuation, which caused luminal narrowing. IVUS images at four levels (a–d) demonstrated the transition from healthy vessel to the site of obstruction (*Figure 2B*, *Video 2*). Intravascular endoscopy corresponding to cross-section c revealed a white intraluminal material, presumed to be residual collagen from the VASCADE MVP device (*Figure 2C*, *Video 3*). Balloon angioplasty was performed using both a semi-compliant 8.0 × 40 mm balloon and a scoring 6.0 × 40 mm balloon (*Figure 3A*). Indentation remained during inflation of both balloons, and the patient experienced localized venous pain during the dilation. Although ∼90% stenosis remained, partial passage of contrast across the treated segment was achieved following the procedure (*Figure 3B*, Supplementary material online, *Video S2*). The occlusion was presumed to result from retained collagen. Since slight symptom improvement was noted after balloon angioplasty, the condition was expected to resolve with natural resorption, and conservative management with compression stockings was selected in consultation with vascular surgery. At the outpatient visit on Day 30, no further symptomatic improvement was observed. However, duplex ultrasonography demonstrated persistent stenosis with partial flow, likely due to both the effect of endovascular treatment and the initial phase of collagen resorption. From Day 35, symptoms began to improve, and at the outpatient visit on Day 55, the swelling had completely resolved. Follow-up duplex ultrasonography at that time confirmed complete resolution of the stenosis.

## Discussion

This is a rare case in which femoral vein occlusion occurred after use of the VASCADE MVP device, with partial improvement following endovascular treatment and complete symptom resolution by 2 months. The putative mechanism was clarified by comprehensive intravascular imaging and close clinical follow-up.

**Figure 4:**
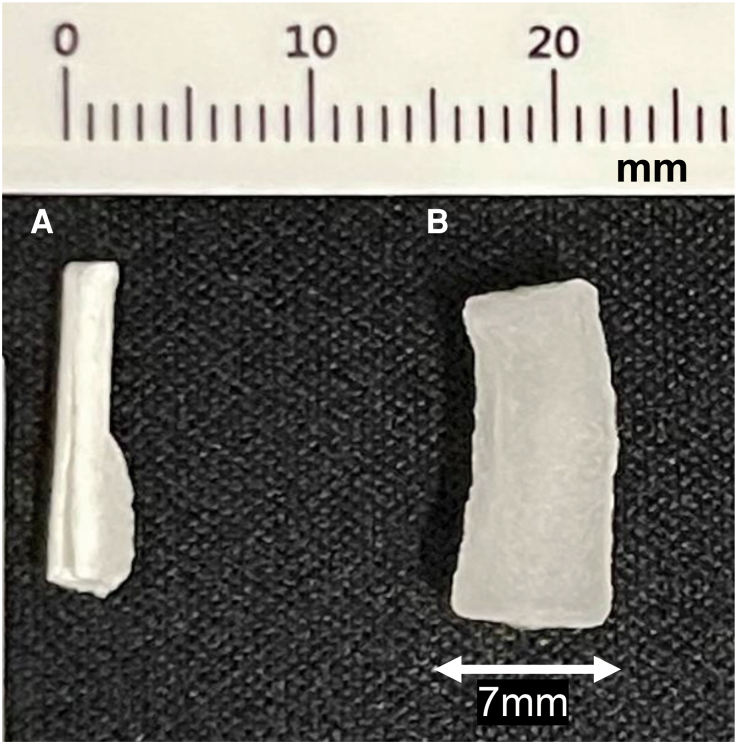
Hydration-induced swelling of the collagen plug used in the VASCADE MVP system. (*A*) The collagen plug before immersion in saline. (*B*) The collagen plug after 30 s of immersion in normal saline, showing expansion to ∼7 mm in width.

The collagen component of VASCADE MVP swells rapidly on contact with fluid and is described in the manufacturer’s instructions for use to expand to ∼7 mm.^7^ In a simple bench observation using normal saline, the plug expanded to ∼7 mm within 30 s, consistent with this description (*Figure 4*). Given that the patient’s common femoral vein (CFV) measured 10.9 × 9.4 mm, inadvertent intravascular retention of the collagen plug could plausibly occlude the lumen. Because this was a single, artificial-condition observation that does not reproduce venous haemodynamics or plasma protein milieu, it should be interpreted as illustrative rather than definitive.

**Figure 5:**
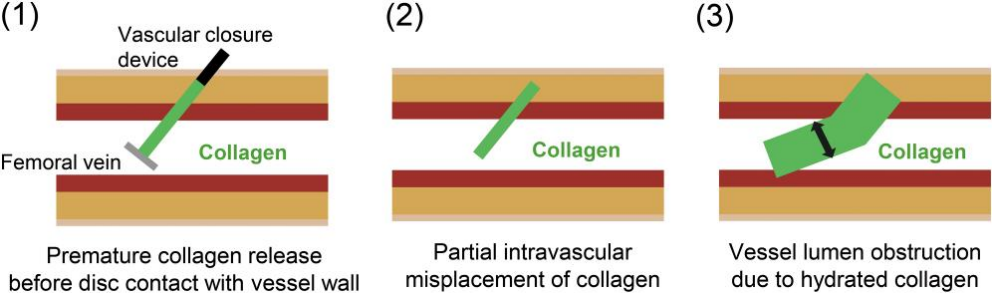
Hypothesized mechanism of femoral vein occlusion due to suboptimal deployment of a vascular closure device. (1) If collagen is exposed prematurely before the intravascular disc fully contacts the vessel wall, part of the collagen may enter the vessel lumen. (2) This can result in partial intravascular misplacement of collagen. (3) Once hydrated, the collagen can expand and mechanically obstruct the vessel lumen.

In light of the complication in the index case, we reconsidered how collagen could be misplaced despite standard technique. By design, VASCADE MVP achieves haemostasis by sequential deployment of an intravascular disc followed by an extravascular collagen plug that seals the puncture tract (see Supplementary material online, *Figure S1*); however, this sequence is reliable only if the disc is fully apposed to the venous wall at the moment of collagen exposure. We therefore hypothesize that premature collagen exposure before complete disc-to-wall contact allowed a portion of collagen to enter the vessel lumen and—once hydrated—expand and mechanically obstruct the lumen, consistent with the failure pathway illustrated in *Figure 5*. Because tactile resistance during disc transition can be mimicked by a venous valve leaflet and temporary surface haemostasis is not definitive, we modified our practice after this event: before collagen exposure, we confirm true disc-to-wall apposition by ultrasonography—obtaining a short-axis view and, on a long-axis view, applying gentle device traction to enhance visualization of persistent linear contact; only after this endpoint is verified do we retract the sleeve to expose collagen (see Supplementary material online, *Figure S2* and Supplementary material online, *Video S3*). To illustrate this checkpoint, Supplementary material online, *Figure S2* and Supplementary material online, *Video S3* show representative images and a clip from a different patient (not the index case).

Beyond deployment technique, consideration of alternative closure strategies is also relevant. Comparative evidence directly contrasting collagen-based and suture-mediated venous closure in electrophysiology or transvenous structural workflows remains limited; however, head-to-head clinical experience in left atrial appendage occlusion suggests that collagen-based closure is a feasible alternative to figure-of-eight sutures with acceptable safety and efficiency profiles.^8^ To fully realize the workflow advantages of collagen-based closure, emphasis should be placed on safe device use and clear response algorithms when complications arise, including early recognition of intraluminal collagen retention and stepwise endovascular management.

To our knowledge, this is the first reported case of venous occlusion caused by a collagen-based haemostatic device. This case is clinically significant as it underscores the importance of proper deployment technique and demonstrates that mechanism-informed conservative management can avert surgery.

## Lead author biography


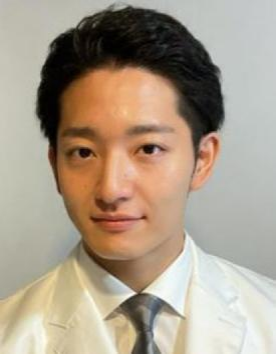


Dr Masaki Honda graduated from Kanazawa University in 2016. After completing his residency in Nagano, he worked in general and cardiovascular medicine at Tokyo Bay Urayasu-Ichikawa Medical Center. In 2024, he joined the Department of Cardiovascular Medicine at Tokyo Medical and Dental University (now Institute of Science Tokyo), focusing on cardiac electrophysiology. He entered its graduate school in April 2025 and is currently engaged in both clinical practice and research on arrhythmias.

## Supplementary Material

ytaf581_Supplementary_Data

## Data Availability

The data underlying this article will be shared on reasonable request to the corresponding author.
