# Supplementary material for: Femoral vein occlusion after VASCADE MVP resolved without surgery: a case report
Source: Eur Heart J Case Rep. 2025 Nov 8;9(12):ytaf581. doi: 10.1093/ehjcr/ytaf581 (PMC12712613; doi:10.1093/ehjcr/ytaf581)
Supplement: ytaf581_Supplementary_Data [file ytaf581_Supplementary_Data.zip › Supplementary Figure legends.docx]

**Supplementary Figure legends**

**Supplementary Figure S1. Step-by-step illustration of the haemostatic mechanism of the VASCADE MVP closure system.**
① The VASCADE MVP is inserted into the introducer sheath, and the disc is deployed.
② The sheath is withdrawn to position the disc against the vessel wall.
③ The sleeve is retracted to expose the collagen plug.
④ The collagen is deployed using the green delivery tube, and the device is removed.

**Supplementary Figure S2.** **Ultrasound of the common femoral vein during haemostasis with VASCADE MVP.**

Representative images from a different patient (not the index case) undergoing haemostasis with VASCADE MVP at the common femoral vein. Panels A (short-axis) and B (long-axis) are shown. The device disc (arrow) appears hyperechoic with acoustic shadowing, and stable apposition to the venous wall prior to collagen exposure is evident.

**Supplementary Video Legends**
**Supplementary Video S1. Pre-treatment distal venography (corresponds to Figure 2A)**Distal contrast injection demonstrating absence of opacification beyond the occlusion. Together with Video 1 (main manuscript), the estimated lesion length is approximately 30 mm. A percutaneous needle marks the skin puncture site under fluoroscopy.

**Supplementary Video S2. Post-treatment venography (corresponds to Figure 3B)**

Distal contrast injection after balloon angioplasty, showing partial passage of contrast across the residual severe stenosis, estimated at approximately 90%, at the treated segment.

**Supplementary Video S3. Long-axis ultrasound of the common femoral vein: gentle device traction enhances visualization of disc–to–venous wall apposition.**Representative clip from a different patient (not the index case). A long-axis view of the common femoral vein shows that gentle traction on the device enhances visualization of the disc firmly apposed to the venous wall.
