# Supplementary figures and images for: Femoral vein occlusion after VASCADE MVP resolved without surgery: a case report
Source: Eur Heart J Case Rep. 2025 Nov 8;9(12):ytaf581. doi: 10.1093/ehjcr/ytaf581 (PMC12712613; doi:10.1093/ehjcr/ytaf581)

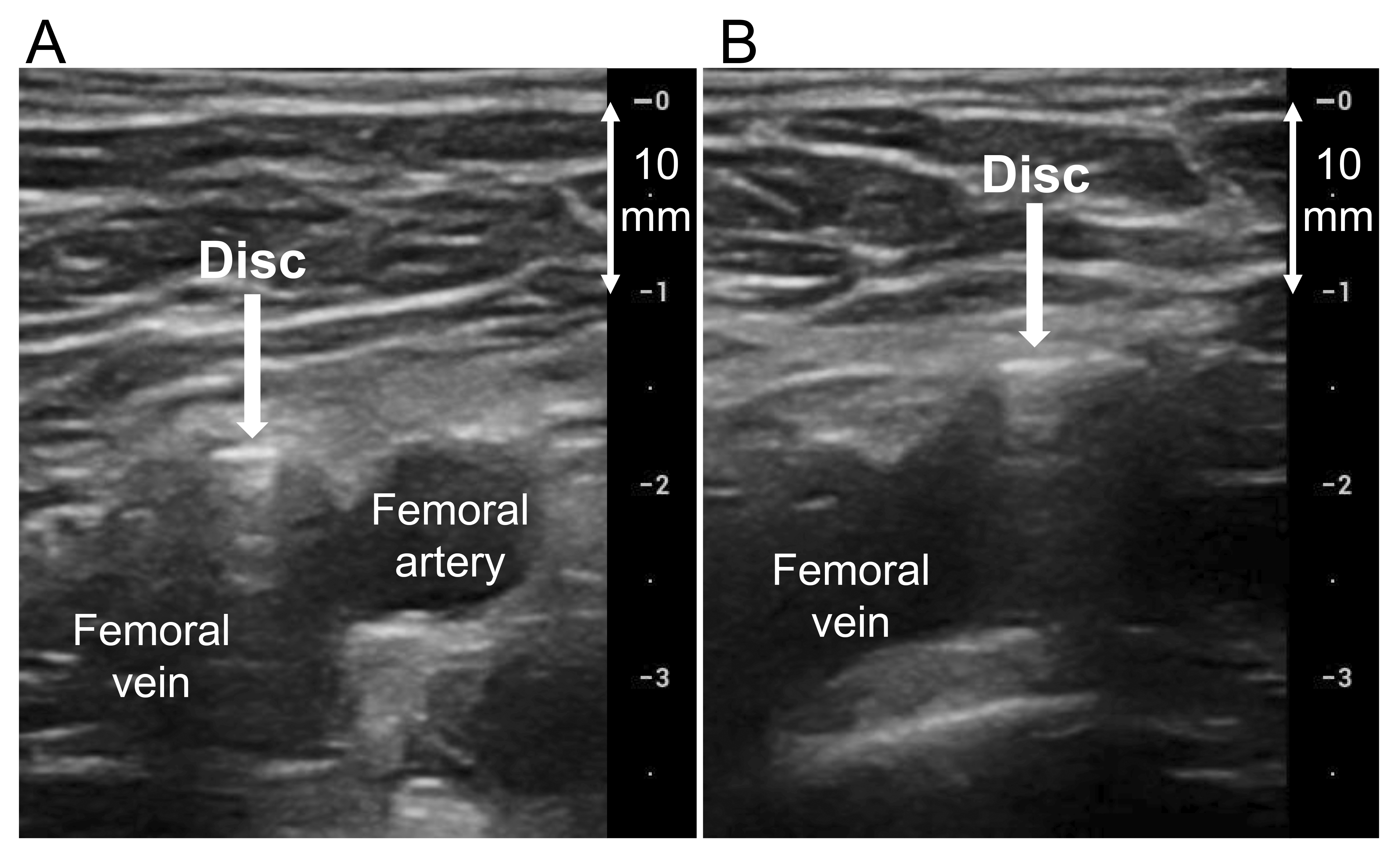

Supplement: ytaf581_Supplementary_Data [file ytaf581_Supplementary_Data.zip › Figure S2_greyscale.tif]

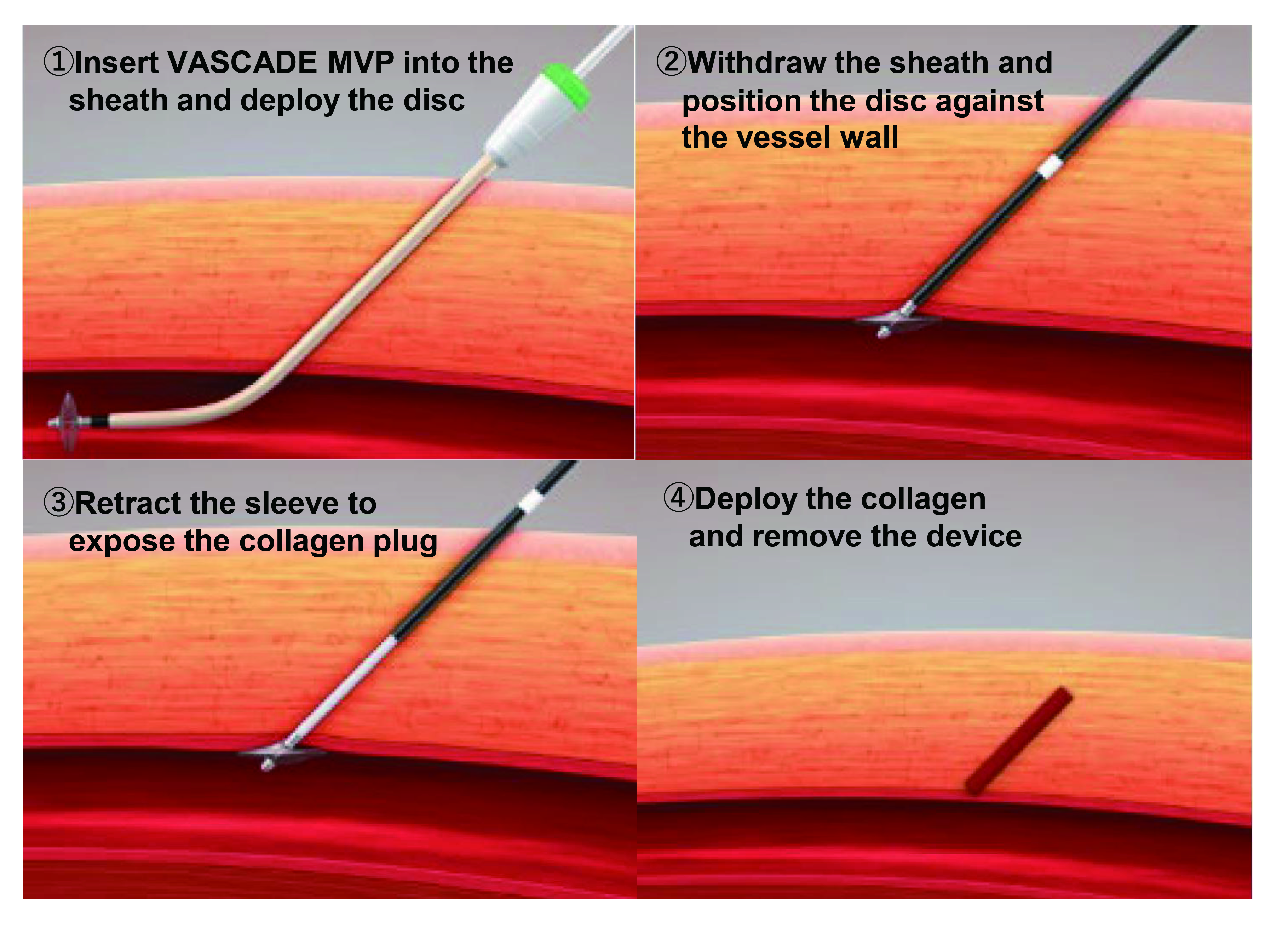

Supplement: ytaf581_Supplementary_Data [file ytaf581_Supplementary_Data.zip › Figure S1_CMYK.tif]
